# Echocardiographic Evaluation of LV Function in Patients with Tachyarrhythmia and Reduced Left Ventricular Function in Response to Rhythm Restoration

**DOI:** 10.3390/jcm10163706

**Published:** 2021-08-20

**Authors:** Christian Schach, Thomas Körtl, Rolf Wachter, Lars S. Maier, Samuel Sossalla

**Affiliations:** 1 Abteilung für Kardiologie, Universitäres Herzzentrum Regensburg, Universitätsklinikum Regensburg, Franz-Josef-Strauß-Allee 11, 93053 Regensburg, Germany; christian.schach@ukr.de (C.S.); thomas.koertl@ukr.de (T.K.); lars.maier@ukr.de (L.S.M.); 2 Klinik und Poliklinik für Kardiologie, Universitätsklinikum Leipzig, Liebigstrasse 20, 04103 Leipzig, Germany; rolf.wachter@medizin.uni-leipzig.de; 3 Klinik für Kardiologie und Pneumologie, Universitätsmedizin Goettingen (UMG), Robert-Koch-Str. 40, 37075 Goettingen, Germany

**Keywords:** atrial fibrillation, tachycardia, ventricular function, speckle tracking, 3D-echocardiography, arrhythmia-induced cardiomyopathy

## Abstract

Aims: Tachyarrhythmia due to atrial fibrillation (AF) is often associated with reduced left ventricular (LV) function and has been proposed to cause arrhythmia-induced cardiomyopathy (AIC). However, the precise diagnostics of AIC and reversibility after rhythm restoration are poorly understood. Our aim was to investigate systolic LV function in tachycardic AF and to evaluate the direct effect of rhythm restoration. Methods: We prospectively studied 24 patients (71% male, age 65 ± 9 years) with tachycardic AF and newly diagnosed reduced left ventricular ejection fraction (LVEF). Just before and immediately after electrical cardioversion (ECV), transthoracic echocardiography was performed. Geometric as well as functional data were assessed. Results: Patients presented with a heart rate (HR) of 117.4 ± 21.6/min and a 2D-/3D-LVEF of 32 ± 9/31 ± 8%. ECV to sinus rhythm normalized HR to 77 ± 11/min with an increase of 2D-/3D-LVEF to 37 ± 9/37 ± 10% (*p* < 0.01 vs. baseline, each). Left ventricular geometry changed with an increase of end-diastolic volume (LVEDV) while end-systolic volume (LVESV) remained unchanged. Parameters concerning myocardial deformation (global longitudinal strain (GLS), strain rate (SR)) decreased whereas the RR interval-corrected GLS (GLSc) remained unchanged. In a simple linear regression model, GLS correlated with 2D- and 3D-LVEF not only before (pre) ECV, but also after (post) ECV. We demonstrate that the increase of LVEF and GLS (ratios pre/post) correlates with the change of HR (ΔHR; *R*^2^ = 0.20, 0.33 and 0.32, *p* < 0.05 each), whereas ratios of GLSc and SR do not significantly correlate with HR (*R*^2^ = 0.03 and 0.01, *p* = n.s. each). Conclusion: In patients with tachyarrhythmia and reduced ejection fraction, ECV leads to immediate improvement in EF and GLS while HR-corrected LV contractility remains unchanged. This suggests that the immediate effects of rhythm restoration are mostly related to changes in left ventricular volume, but not to an acute improvement of heart-rate independent contractility.

## 1. Introduction

Atrial fibrillation (AF) is the most frequent rhythm disorder and is often associated with heart failure (HF). These two conditions themselves share common risk factors [1]. In addition to that they also bear a certain conjunction in terms of origin and effect. Proceeding HF frequently leads to AF, whereas AF, especially tachycardic, may lead to HF in the form of arrhythmia-induced cardiomyopathy (AIC). Up to every fourth patient with AF develops tachycardia [2]. The establishment of sinus rhythm (SR) is a major therapeutic concept in most patients with AF, especially if HF coexists. It has been shown that an early rhythm-control therapy is associated with a lower risk of adverse cardiovascular outcomes including less hospitalizations due to worsening of heart failure [3]. Limited evidence suggests that ventricular rate during AF does not predict reversibility of HF [4]. AF ablation trials have questioned that rate control compared to rhythm control is equally effective for the recovery of ventricular function in patients with paroxysmal/persistent AF and associated HF [5,6]. The significant improvement of LV function and HF symptoms after AF ablation (compared to medical rate control) supports the premise that AF alone can lead to systolic HF despite appropriate rate control. Electrical cardioversion (ECV) of AF to sinus rhythm has been shown to increase LVEF within 1–3 days [7,8,9]. Alterations in LV contractility during AF—which normalize more or less quickly after conversion to SR—may not be detected when the interval between conversion/cardioversion and echocardiography is too long. Thus, we intended to measure systolic LV function immediately after conversion into SR.

Besides LV ejection fraction (LVEF), which remains the most commonly used conventional parameter, we employed speckle-tracking echocardiography (STE) and global longitudinal strain (GLS) analysis, as they are of high resolution. In addition to that, they have been validated in comparison to magnetic resonance and sonomicrometry [10,11,12]. The high resolution is especially helpful in tachycardia. This phenomenon was shown in a study examining inappropriate sinus tachycardia [13], which makes this technique attractive for evaluation of myocardial function in patients with AF [14,15]. However, differences in heart rate (HR), or more specifically RR interval, may aggravate the interpretation of echocardiographic parameters, especially of systolic LV-function. For this reason, we used the concept of frequency correction, proposed by Olsen et al. [16]. This method was proved to be simple, reliable and of clinical relevance [14]: GLS divided by the square root of the RR interval in seconds gives the RR interval-corrected global longitudinal strain (GLSc).

We hypothesized that ECV leads to immediate improvements in LVEF by a combination of volume-dependent effects (e.g., increase in preload by higher left ventricular volume) and direct effects on left ventricular contractility. Therefore, geometric as well as functional parameters were comprehensively recorded using echocardiography directly before and after energy application in order to only detect the remodeling-independent effects of AF.

## 2. Methods

### 2.1. Study Population

**Figure 1:**
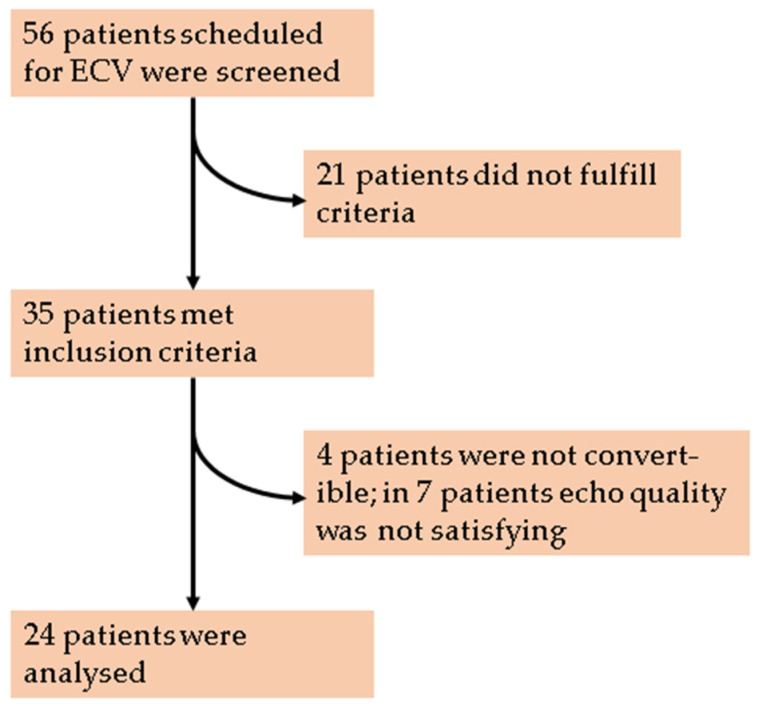
Patient flowchart. ECV: Electrical Cardioversion.

This prospective observational single center study was reviewed by the independent ethics committee of the University of Regensburg (ethical approval 18-1072-101) and all patients provided written informed consent. We included patients with tachycardic AF and newly diagnosed reduced LVEF < 50%, who were scheduled for ECV. If the intake of oral anticoagulation (OAC) in the past three weeks was uncertain, a transesophageal echocardiography was performed to rule out intracardiac thrombus. Patients with known left ventricular systolic dysfunction, more than moderate valvular heart disease and patients with acute coronary syndrome within in the last three months were excluded. All patients underwent echocardiography just before and after ECV. Patients with unstable clinical conditions, suboptimal image quality for analysis (defined as more than three poorly visualized segments in the apical view) or arrhythmias other than AF were excluded (see Figure 1).

Patients scheduled for ECV were screened for HR > 100/min and LVSD (EF < 50%). Thirty five patients met the inclusion criteria. Four of them could not be cardioverted, echocardiographic image quality in seven patients was not satisfying (more than three of 18 poorly visualized segments in the apical view). Twenty four patients were analyzed. 

### 2.2. Echocardiography and Data Analysis

**Figure 2:**
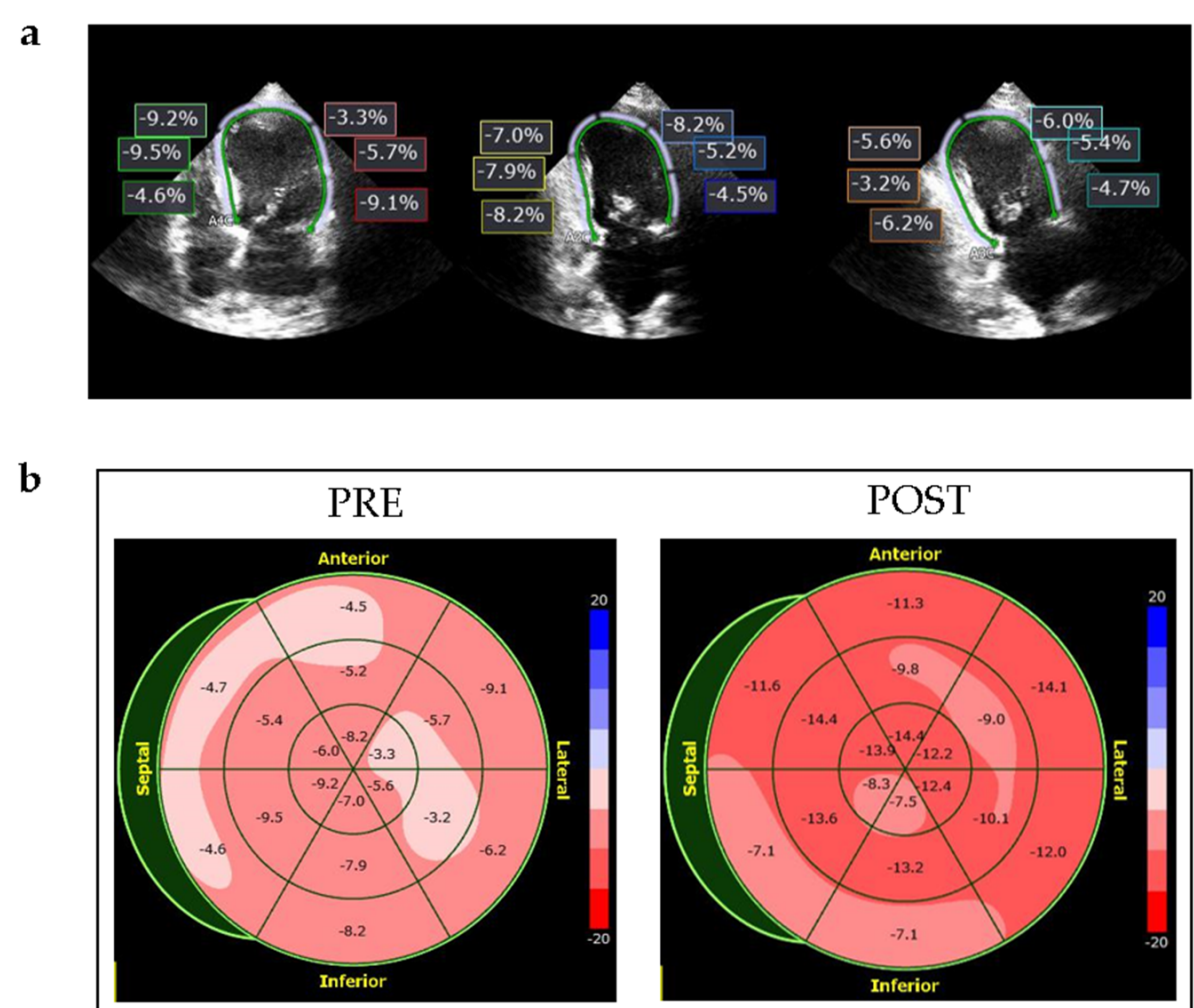
Left ventricular strain in a patient with tachycardic AF and reduced EF. (**a**) Two-dimensional echocardiographic images showing strain measures of the left ventricle before electrical cardioversion (ECV) in the apical four, three and two chamber view from left to right; (**b**) 18 segment bull’s eye plots of this patient before (pre) and after (post) ECV. AF: atrial fibrillation; EF: ejection fraction.

Echocardiographic images were obtained using a Philips Epiq CVx ultrasound scanner (Philips, Hamburg, Germany) with a transthoracic matrix phased-array transducer (X5-1, Philips xMatrix probe, 5-1 MHz). Standard 2D views in the parasternal and LV apical sound field were used, frame rate > 50 frames/s. Two-beat 3D full LV volumes were acquired for 3–5 cycles in AF and 2–3 cycles in sinus rhythm. Image acquisition occurred at a frame rate of 26.5 ± 3.4 30 frames/s. Careful attention was paid to adjusting the sector width and depth to include the entire LV myocardium, including the epicardial surface within the pyramidal scan volume. All original echocardiographic images were captured with the same machine, probe and investigator. Acquired 2D and 3D data were digitally transferred to a separate workstation for offline analysis of LV volumes, LVEF and strain using the IntellisSpace Cardiovascular Software (Cardiovascular Image and Information System Management System, Philips Medical Systems, Best, The Netherlands) by a blinded user. Image loops (three in AF, one in sinus rhythm) with the best image quality were used for analysis. Seven patients with suboptimal image quality, defined as more than three (out of 18, compare Figure 2) poorly visualized segments in the apical view were excluded (Figure 1). Measurements were performed semi-automated. All 18 segments were averaged for calculation of GLS and SR. End-systolic and end-diastolic LV volumes obtained from the apical four-, two- and three- chamber views were calculated using the method of disks, and LVEF obtained by 2D echocardiography was calculated using Simpson’s biplane and the Teichholz method. 

### 2.3. Electrical Cardioversion and Anesthesia

Anesthesia was done via sevofluran inhalation (8 vol%) until loss of consciousness. Blood pressure, heart rate and oxygen saturation were monitored. Synchronized shocks to a maximum of three shocks (300-360-360 J, biphasic) were delivered until sinus rhythm was restored [17]. All shocks were delivered using Lifepak 15 (Stryker/Physio-Control Inc., Redmond, DC, USA) through self-adhesive electrodes. All shocks were administered with the electrodes placed in anterior–posterior position [18]. If sinus rhythm could not be established after the third energy application, patients were excluded from the study. Mean time of anesthesia was 2.5 ± 1 min. 

### 2.4. Statistical Analysis 

Continuous variables are expressed as mean ± SD, categorical variables as percentages. Due to the nature of the study, pre vs. post ECV comparisons were made by paired Student’s t-testing. Relationship analysis between ΔHR and strain measurements as well as LVEF were assessed using a simple linear regression analysis and using Pearson’s correlation. Comparison of the ratios (Figure 4e) was done by one-way ANOVA Tukey’s multiple comparison test. *p* values < 0.05 were considered statistically significant. Data were analyzed using standard statistical software (Graphpad Prism, version 9, San Diego, CA, USA).

## 3. Results

### 3.1. Study Population and Baseline Characteristics

Thirty five patients were screened, but 11 patients had to be excluded from analysis (four not convertible, seven unsatisfying image quality, see Figure 1). Clinical characteristics of the 24 patients are given in Table 1: Three quarters of patients were on OAC and 62.5% on beta blockers. One patient was on digoxin (4.2%), two patients received amiodarone (8.4%) (see medication regime in Table 1). Patients were at high risk for stroke (CHA_2_DS_2_-VASc-Score 3.1 ± 1.4). Patients who were not on OAC at presentation received initiation of OAC, and every patient whose intake of OAC within the last three weeks was uncertain received a transesophageal echocardiography to rule out intracardiac thrombus. LVEF was severely reduced (32.5 ± 9.1%) ranging from 13.8% to 46% and HR_pre_ ranged from 63/min to 165/min. After ECV, systolic blood pressure increased from 134 ± 18 mmHg to 138 ± 16 mmHg without reaching the significance level (*p* = 0.07), diastolic blood pressure remained in a similar range of 81 ± 10 mmHg (pre) and 78 ± 11 mmHg (post; *p* = 0.17).

### 3.2. LVEF Correlates with GLS Pre and Post Electrical Cardioversion

**Figure 3:**
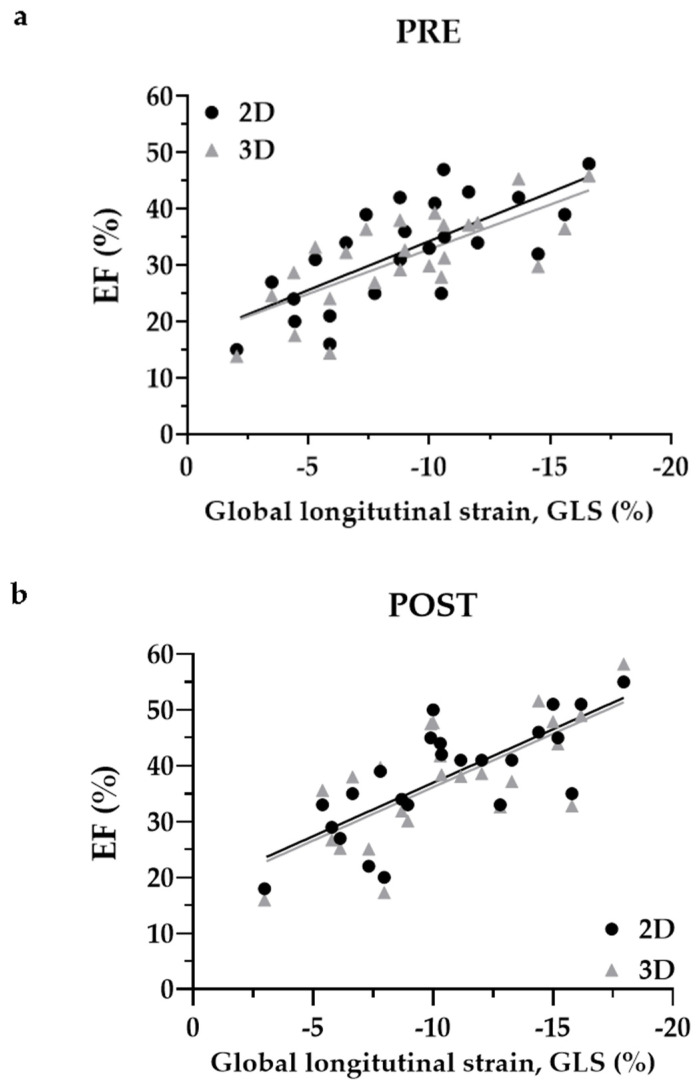
Scatter plots comparing GLS with EF. GLS correlates with 2D and 3D left ventricular EF in a model of simple linear regression before ((**a**), pre) and after ((**b**), post) ECV. The slopes deviate significantly from zero and do not differ significantly among each other. GLS, global longitudinal strain; EF, ejection fraction; ECV, electrical cardioversion.

LV function measured by Simpson’s method (2D) and by 3D volumetry correlates with global longitudinal strain before (pre) and after (post) cardioversion in a model for simple linear regression. More negative values of GLS represent more strain of the LV (Figure 3). Slopes for 2D- and 3D-LVEF pre cardioversion deviate significantly from zero (*p* < 0.001 for 2D-LVEF vs. GLS and for 3D-LVEF vs. GLS) with a goodness of fit (*R*^2^) of 0.52 for 2D- and 0.54 for 3D-LVEF, thus there is a significant correlation between 2D/3D-LVEF and GLS (Figure 3a). Among each other, slopes did not differ significantly (−1.74 ± 0.36 vs. −1.59 ± 0.31, *p* = 0.76; pooled slope = −1.66). Post cardioversion slopes did also deviate significantly from zero (each *p* < 0.001) with an *R*^2^ of 0.57 and 0.51 for 2D- and 3D-LVEF (Figure 3b). Since the slopes were not different either (−1.91 ± 0.35 vs. −1.91 ± 0.39; *p* = 0.99), a pooled slope of −1.91 can be specified. Moreover, slopes for 2D- (pre vs. post) and 3D-LVEF (pre vs. post) did not differ. These results indicate that the measurement of 2D- and 3D-LVEF is reliable in these patients and GLS correlates with LVEF, which has been shown in normofrequent patients before [12]. ECV did not influence the correlation between EF and GLS. 

### 3.3. Acute Effects of ECV on Geometric and Functional Parameters

**Figure 4:**
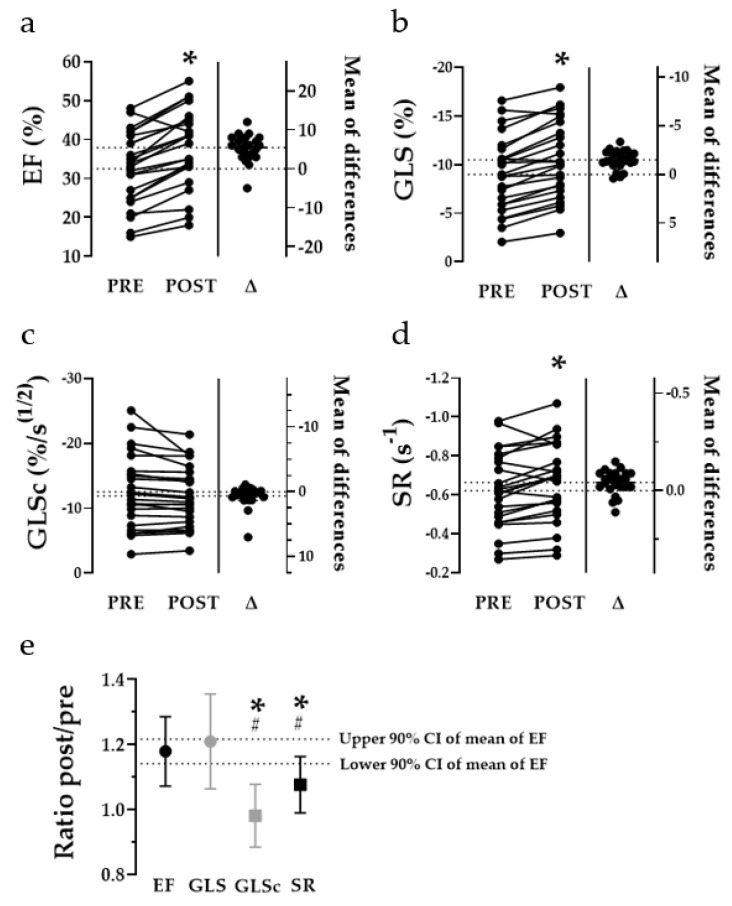
Measures of systolic LV function. Estimation plots showing an increase of EF, GLS and SR pre vs. post ECV (**a**,**b**,**d**), whereas GLSc showed no significant difference (**c**). (**e**) Scatter plot of the ratios (post/pre) of EF, GLS, GLSc and SR. * indicates *p* < 0.05 vs. pre (**a**,**b**,**d**) and vs. EF (**e**), ^#^ indicates *p* < 0.05 vs. GLS (**e**). EF, ejection fraction; GLS, global longitudinal strain; SR, strain rate; ECV, electrical cardioversion; GLSc, global longitudinal strain corrected for RR interval.

Conversion to sinus rhythm led to a mean decrease of HR of 40.5 ± 18.9/min and an immediate increase of 2D-LVEF (Figure 4a), 3D-LVEF and LVEF calculated by the Teichholz equation of 5.4 ± 3.4%, 5.9 ± 4.1% and 6.5 ± 9.7% (Table 2). End-diastolic diameter and volumes increased, whereas there was no significant difference in end-systolic values. The size of the left atrium remained unchanged. GLS showed a decrease (more strain) of −1.51 ± 0.9% and a strain rate of −0.05 ± 0.6 s^−1^ (Table 2; Figure 4b,d). RR-corrected GLS (GLSc) did not differ pre vs. post ECV (Figure 4c). The ratios (post/pre) for LVEF (1.18 ± 0.10) and GLS (1.21 ± 0.14) differ, in contrast to the HR-independent parameters GLSc (0.98 ± 0.10) and SR (1.08 ± 0.09), significantly from zero (Figure 4e). 

### 3.4. Delta HR-Associated Increase of Ejection Fraction vs. Contractility

**Figure 5:**
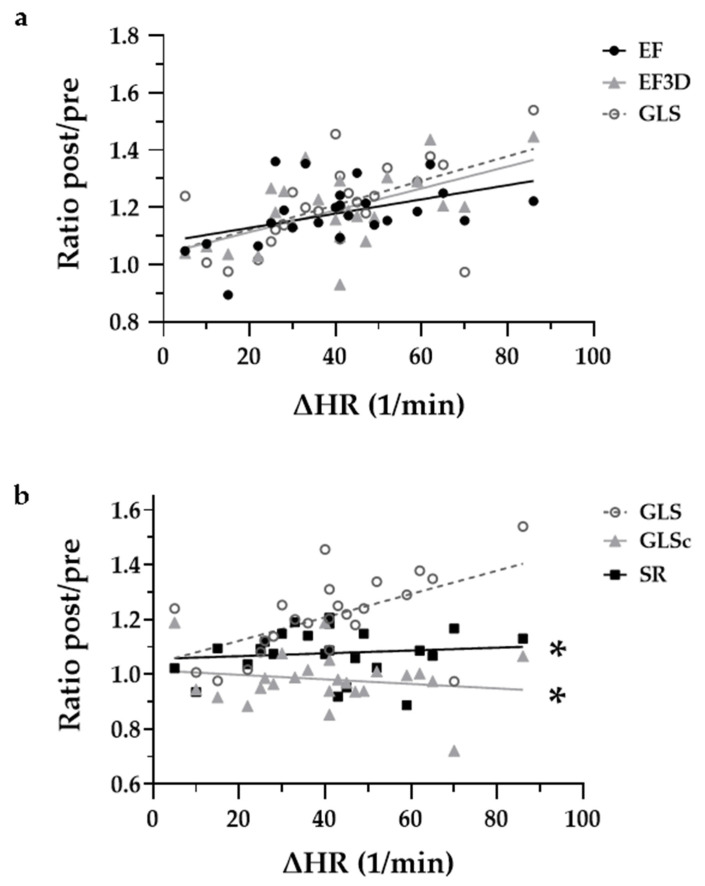
Influence of HR on systolic LV function (given as ratio post/pre ECV). Scatter plotting ΔHR vs. change of measures for systolic LV function calculated as ratios post/pre for EF, EF3D, GLS (**a**), GLS, GLSc and SR (**b**). Slopes in a simple linear regression for EF, EF3D and GLS significantly differ from zero, but are not significantly different (**a**). Slopes for GLSc and SR do not deviate significantly from zero and both differ significantly from GLS (**b**). ΔHR, difference of heart rate before and after electrical cardioversion; LV, left ventricular; EF, ejection fraction measured via 2D; EF3D, ejection fraction measured via 3D; GLS, global longitudinal strain; GLSc, global longitudinal strain corrected for RR interval; SR, strain rate. * indicates a *p*-value of <0.05 vs. slope GLS.

The means of 2D- as well as 3D-LVEF were higher after (post) than before (pre) ECV (Figure 4a, Table 2). To determine the extent of the increase, ratios (pre/post) were calculated. These ratios of 2D- as well as 3D-LVEF and GLS increased with ΔHR (HR_pre_ − HR_post_) showing a positive correlation (slopes deviate significantly from zero, *p* < 0.05 each; R^2^ for ratios of 2D-, 3D-LVEF and GLS were 0.20, 0.32 and 0.31 and Pearson’s r vs. ΔHR were 0.45, 0.57 and 0.56, which are shown in Figure 5a). Slopes of the simple linear regression models for the ratios of LVEF vs. ΔHR and GLS vs. ΔHR are not significantly different (*p* = 0.54), the pooled slope equals 0.003 (Figure 5a). For better visual comparison, data points of ratio-GLS vs. ΔHR were transferred to Figure 5b and compared with ratio-GLSc and ratio-SR. Ratios of GLSc and SR showed no increase with ΔHR as the slopes of their simple linear regression model do not deviate significantly from zero (*p* = 0.10 for GLSc and *p* = 0.59 for SR). *R*^2^ for the ratios of GLSc and SR are 0.03 and 0.01; Pearson’s was −0.17 for ratio-GLSc vs. ΔHR and 0.11 for ratio-SR vs. ΔHR. Moreover, ratio-GLSc- and ratio-SR-slopes differ significantly from ratio-GLS (−0.0008 ± 0.001 vs. 0.004 ± 0.001, *p* < 0.01 and 0.0005 ± 0.001 vs. 0.004 ± 0.001, *p* < 0.05). There was no significant difference in ratio-GLSc- and ratio-SR-slopes (*p* = 0.34, pooled slope −0.0001). This means that GLS, but not GLSc and SR, correlate with ΔHR, and rhythm control via ECV did not impact these parameters of frequency corrected contractility.

## 4. Discussion

HF and AF very often coexist. The recognition concerning arrhythmia-induced or arrhythmia-mediated cardiomyopathy is growing. However, the detection of LV function remains a difficult issue in patients with AF especially when associated with tachycardia. It is possible that the extent of contractile dysfunction is sometimes overestimated in the presence of atrial tachyarrhythmia. We sought to separate acute adaptive LV contractile changes due to tachyarrhythmia from distinct LV remodeling processes. The latter ones may recover if sinus rhythm persists. The discrimination of adaptive LV function vs. LV remodeling plays a relevant role for daily practice but also for further clinical trials. This study was designed as a single center study, thus the sample size is rather small, which is a limitation of the study.

Our data indicate that 2D-, 3D-LVEF, as well as LV strain, improved immediately after ECV. This improvement was dependent on the degree of HR-reduction. Remarkably, measures more specific for contractility (i.e., SR and GLSc) than LVEF did not display this frequency dependence. These findings are important for the interpretation of data in the literature and for the design of clinical trials in the field of AIC. Raymond et al. showed that LVEF (echocardiographically measured) increased by 10% up to 61% two to 24 h after cardioversion. Heart rates were 77/min before and 59/min after ECV [9]. A recent study by Müller-Edenborn et al. reports an increase in LVEF of 13% (MRI and echocardiography) within 3 days after ECV in patients with otherwise unexplainable systolic LV dysfunction [7]. Baseline heart rate was 107.5 ± 20.9/min and thus better comparable to our study (mean HR_pre_ 117.4 ± 21.6/min). However, this study did not report LV geometry before and after ECV within the same modality. In the former study, an increase of LVEDV after ECV could be detected; at that time, there was no relining by 3D-data. In AF-ablation studies, end-diastolic LV size declined after intervention: end-systolic diameters decreased after 1–12 months, while LVEF increased after 1–6 months post procedure [19]. Likewise, LVESVi and LVEDVi decreased 6 months post ablation in the CAMERA-MRI study, which evaluated the recurrence of AF after ablation compared to drug therapy [5]. The time course of LVEF recovery over several months suggests a prolonged “healing” from systolic heart failure, even if there is a quick initial LVEF improvement within the first weeks after ECV [20,21]. Baseline heart rates were 72/min (rate control group) and 103/min (inadequate rate control group) in Hsu et al. [19] and 79/min in Prabhu et al. [5]. However, AIC cannot be excluded as on origin for heart failure in all these studied collectives, thus an AIC in some of these patients remains feasible.

Our data show an acute increase of LVEDD and LVEDV, whereas end-systolic parameters remained unchanged. This might be due to prolonged diastolic filling after rhythm control and HR reduction by ECV. A study using cardiac scintigraphy with technetium-99m-labeled red blood cells demonstrated a strong correlation of RR interval with LVEF in patients with AF [22]. An increase in preload (LVEDV), which is the consequence of prolonged LV filling, is known to translate into a gain in contractility via the Frank–Starling mechanism. We additionally employed strain measurements in order to further characterize LV motion. Global longitudinal strain (GLS) has proven to be as accurate as 2D and 3D echocardiography in the quantification of LV function [23]. Furthermore, it bears some advantages in tachycardic heart rates due to its high spatial resolution [11,13]. As expected, GLS behaved similarly to LVEF pre and post ECV in our study. Due to the fact that LVEF should be critically used as a marker for LV contractility without considering the LV loading situation [24], we deployed the measurement of strain rate (SR), which is less load dependent than strain [25]. Furthermore, SR proved to be independent from acute alterations of HR in right atrial paced pigs [26]. Like SR, heart rate corrected GLS (GLSc) showed no correlation with the change of HR in our analysis (compare Figure 5b). In light of our findings, previous studies examining the acute effect of ECV on LV systolic function need to be reconsidered, as rhythm control itself may lead to an increase in LVEF if accompanied by prolongation of diastolic filling [7,8,9]. The closer the measurement of LVEF is to rhythm control, which has been the case in the literature at least 1–2 h pre and 1–2 h post ECV [27], the more important the consideration of HR becomes. From a teleological point of view, the decrease of EF in tachycardia, as often seen in AF, can be considered a protective reduction of cardiac output. However, as the main clinical implication of this study, the estimation of LV function via measurement of EF may lead to an overestimation of systolic heart failure. Hence, for the assessment of LV systolic function, we suggest the measurement of LVEF in normofrequency. As an alternative, the application of the frequency independent parameters of left ventricular function GLSc and SR may serve as a reliable measuring tool of systolic LV function, especially if patients, whose heart rates are not yet controlled properly, are examined.

## 5. Summary and Conclusions

Here we show that the restoration of normofrequent sinus rhythm immediately improves LVEF and global longitudinal strain. Both parameters were dependent on the extent of HR reduction, but not for the HR independent SR or the heart rate corrected GLSc parameter.

In summary, tachycardic AF is a condition in which systolic left ventricular function is reduced, which is—dependent on the study design—more or less visible. The increased end-diastolic volume can be explained by a prolonged diastolic filling period as a direct consequence of rhythm restoration. Moreover, an increased preload following rhythm restoration may potentially improve contractility via the Frank–Starling mechanism, leading to the improvement of parameters measuring LV deformation and ejection.

In conclusion, we recommend deploying parameters of systolic LV function other than LVEF (i.e., SR and GLSc) in daily clinical practice but also in clinical trials if neither rhythm nor frequency control can be achieved.

## Figures and Tables

**Table 1:** Clinical Baseline Characteristics.

Clinical Characteristic	*n* = 24
Age	65.6 (9.0)
Gender, male	17 (70.8)
Weight (kg)	86.5 (18.6)
Height (cm)	171.0 (8.9)
BMI (kg/m^2^)	29.4 (5.3)
BSA (m^2^)	2.0 (0.3)
Diabetes	6 (25.0)
Arterial hypertension	17 (70.8)
Renal insufficiency	5 (20.8)
CHA_2_DS_2_-VASc-Score	3.1 (1.4)
Smoker status	6 (25.0)
Hyperlipidemia	13 (54.2)
NYHA class	2.6 (0.7)
Coronary artery disease (%)	11 (45.8)
Myocardial revascularisation by CABG	3 (12.5)
Aortic valve replacement	2 (8.3)
Betablocker	15 (62.5)
ACEI	14 (58.3)
Mineralocorticoid antagonist	4 (16.7)
Calcium antagonist	12 (50.0)
Diuretics	11 (45.8)
Digoxin	1 (4.2)
Amiodarone	2 (8.3)
OAC	18 (75.0)

Continuous variables are presented as mean (SD), categorical as *n* (%). NYHA, New York Heat Association; CABG, coronary arterial bypass graft; ACEI, angiotensin converting enzyme inhibitor; OAC, oral anticoagulation; BMI, body mass index; BSA, body surface area.

**Table 2:** Comparison of Echocardiographic Parameters Pre and Post Electrical cardioversion.

Echocardiographic Parameter (*n* = 24)	Pre	Post	*p*-Value
HR (1/min)	117.4 (21.6)	76.9 (11.5)	<0.001
R-R Interval (ms)	531 (120)	796 (110)	<0.001
LVEF, Simpson (%)	32.5 (9.1)	37.9 (9.8)	<0.001
LVEF, 3D (%)	31.2 (8.2)	37.1 (10.3)	<0.001
LVEF, Teichholz (%)	30.1 (9.1)	36.6 (10.7)	0.004
LVEDD (mm)	50.7 (8.0)	52.6 (7.2)	<0.001
LVESD (mm)	43.5 (7.9)	43.2 (6.8)	0.698
LVEDV (mL)	143.9 (49.4)	154.3 (48.1)	<0.001
LVESV (mL)	101.1 (42.5)	99.4 (41.5)	0.127
LVEDVi (mL/m^2^)	70.5 (21.2)	75.8 (20.3)	<0.001
LVESDVi (mL/m^2^)	49.3 (18.0)	48.6 (17.8)	0.220
LA, area (cm^2^)	27.3 (3.4)	28.1 (4.4)	0.215
LAVi (mL/m^2^)	51.2 (10.2)	52.9 (12.2)	0.181
GLS (%)	−8.9 (3.8)	−10.5 (3.9)	<0.001
GLSc (%/s^(1/2)^)	−12.5 (5.5)	−11.9 (4.5)	0.057
SR (%)	−0.62 (0.19)	−0.67 (0.19)	0.002

Functional and geometric echocardiographic parameters before (pre) and after (post) electrical cardioversion.HR, heart rate; LVEF, left ventricular ejection fraction; LVEDD, left ventricular end-diastolic diameter; LVESD, left ventricular end-systolic diameter; LVEDV, left ventricular end-diastolic volume; LVESV, left ventricular end-systolic volume; LVEDVi, left ventricular end-diastolic volume indexed to body surface area; LVESVi, left ventricular end-systolic volume indexed to body surface area; LA, left atrial area; LAVi, left atrial volume indexed to body surface area; GLS, global longitudinal strain; GLSc, global longitudinal strain corrected by the square root of the R-R interval; SR, strain rate. Variables are presented as mean (SD). *p*-values are calculated by the Student’s *t*-test.

## Data Availability

The data presented in this study are available on request from the corresponding author. The data are not publicly available to ensure that they are only used for research purposes.
